# Feasibility of systemic therapy in unresectable gastric/gastroesophageal junction cancer with overt bleeding

**DOI:** 10.1016/j.apjon.2025.100750

**Published:** 2025-07-07

**Authors:** Yanhong Yao, Zhentao Liu, Hua Zhang, Xinhua Shi, Fangfang Huang, Yi Zhang, Lu Chen, Yanyan Shi, Baoshan Cao

**Affiliations:** a Department of Medical Oncology and Radiation Sickness, Peking University Third Hospital, Beijing, China; b Research Center of Clinical Epidemiology, Peking University Third Hospital, Beijing, China

**Keywords:** Gastric cancer, Gastroesophageal junction cancer, Overt bleeding, Anti-cancer therapy

## Abstract

**Objective:**

To evaluate the feasibility of modified systemic anti-cancer therapy in unresectable gastric cancer or gastroesophageal junction cancer (GC/GEJC) patients with overt bleeding (OB).

**Methods:**

This retrospective study included individuals with unresectable GC/GEJC and OB who received systemic anti-cancer therapy. Treatment feasibility was assessed. Risk factors for persistent overt bleeding post systemic therapy (POBPST) were explored.

**Results:**

Among the 52 individuals included, 19 (36.5%) experienced active OB within one month prior to initiating anti-cancer therapy, while 33 (63.5%) did not. Hemostasis was achieved via endoscopic intervention in 2 patients and conservative medical management in 17. A total of 5 patients received immune checkpoint inhibitor monotherapy, and 47 received chemotherapy-based systemic therapy, with 70.2% (33/47) requiring a 25% dose reduction. Among those with active OB, 73.7% (14/19) received intravenous therapy, with a median interval of 16 days (range: 3–25) from hemostasis to treatment initiation. The overall objective response rate (ORR) and disease control rate (DCR) were 42.3% and 90.4%, respectively. Median progression-free survival (mPFS) and overall survival (mOS) were 10.3 and 16.1 months. POBPST occurred in 28.8% of individuals and was associated with poorer survival. Grade 3–4 treatment-related adverse events occurred in 36.5% of patients. Multivariate analysis identified a Modified Barthel Index (MBI) score < 75 and disease progression or stable disease as independent risk factors for POBPST.

**Conclusions:**

With appropriate hemostatic management, modified dose systemic anti-cancer therapy was feasible and generally well tolerated in patients with unresectable GC/GEJC and OB. Nurses’ role was indispensable for this high-risk population.

**Trial registration:**

The study has been registered in clinical trials.gov (NCT06522542).

## Introduction

The incidence and mortality of gastric cancer were ranked top five for cancer worldwide in 2020.^1^ Gastric cancer or gastroesophageal junction cancer (GC/GEJC) was a heavy burden in China.^2^ Hemorrhage was a common complication and may influence the treatment for GC/GEJC.^3^

Radical gastrectomy was effective for surgical GC/GEJC patients with bleeding,^4^ but not available for local advanced or distant metastatic patients. Local therapy strategies those had both hemostatic and anti-cancer effects, such as transcatheter arterial embolization and radiotherapy, can control the tumor within a certain period, but rebleeding was common and the survival was poor.^5^, ^6^, ^7^ Systemic therapy was one of the main anti-cancer strategies^8^ for unresectable GC/GEJC patients. However, bleeding not only increased the patients’ suffering but also affected the anti-cancer therapy for unresectable patients, due to worry about worsening bleeding and shortening survival.^7^^,^^9^ There were almost no reports on efficacy and safety of chemotherapy-based systemic treatment for unresectable GC/GEJC patients with overt bleeding (OB).

Previously, our study demonstrated that the OS for GC/GEJC patients with OB who receiving systemic treatment was similar to that for those without OB.^10^ Which systemic treatment regimen should be chosen for GC/GEJC patients with OB? The GO2 study reported that older and/or frail advanced gastroesophageal cancer patients receiving reduced-intensity chemotherapy experienced a better quality of life and noninferior survival compared to those receiving standard chemotherapy.^11^ Another study found that grade 2–4 anemia for malignant tumor patients increased the risk of dose delay or reduction in subsequent chemotherapy cycles compared to grade 1 or no anemia.^12^ These reports give us the signal that chemotherapy dose modification for frail patients or with grade 2–4 anemia may be a better choice.

Besides, oncology nurses had made irreplaceable and crucial contribution in cancer control plans, especially for frail patients and patients with comorbidities.^13^ Nursing interventions had a significant positive physical and psychological effect on health literacy and quality of life.^14^^,^^15^ Nursing plays an important role in the treatment and evaluation for GC/GEJC patients with OB. The study collected the data of unresectable GC/GEJC patients with OB who received standard or modified systemic treatment as initial anti-cancer therapy by both physicians and nurses retrospectively. We investigated the manifestation and hemostatic therapy of the initial OB and persistent overt bleeding post systemic therapy (POBPST), evaluated the effectiveness and safety of modified systemic anti-cancer therapy for patients with OB, and assessed the risk factors of POBPST from the perspectives of nurses and doctors respectively.

## Methods

### Patients

This retrospective study enrolled GC/GEJC patients who presented with OB from January 1, 2013 to December 31, 2024 at Department of Medical Oncology and Radiation Sickness of Peking University Third Hospital. The inclusion criteria were as follows: (1) aged 18 years and older, (2) histologically diagnosed as GC/GEJC, (3) evaluated as unresectable GC/GEJC by surgeons, (4) accompanied by OB at initial diagnosis of GC/GEJC, (5) Eastern Corporative Oncology Group Performance Status (ECOG-PS) 0–2, (6) received at least one dose of systemic treatment, including chemotherapy-based regimens or immune checkpoint inhibitors (ICIs), as an initial anti-cancer regimen within one month of OB, (7) with no active OB or active OB had been controlled before systemic treatment, (8) hemoglobin (Hb) level higher than 80 g/L for chemotherapy and 60 g/L for ICIs monotherapy before systemic treatment, and (9) followed up for a minimum of three months. The exclusion criteria included (1) received gastrectomy before systemic therapy, and (2) had no available clinical data.

### Data collection and outcome evaluation

Physicians collected clinical data, including basic message, disease history, clinical stage according to the eighth edition of American Joint Committee on Cancer (AJCC) cancer staging manual, treatment regimens, tumor response to therapy, treatment-related adverse events (TRAEs), etc. Nurses collected patient status assessment data including ECOG-PS, body mass index (BMI), activity of daily living (ADL) evaluated by Modified Barthel Index (MBI) scores,^16^^,^^17^ and Morse Fall Scale.^18^^,^^19^ MBI scores and Morse Fall Scale were translated into Chinese (Supplementary File 1 and Supplementary File 2).

OB was defined as (1) hematemesis, melena, hematochezia, or (2) gastroscopy visible bleeding due to GC/GEJC. OB was recognized as stopped when the above two situations disappeared. Active OB was severe hemorrhage that was defined as meeting at least one of the following criteria: (1) hematemesis, (2) continuously worsening melena (increasing by 30% per day), or hematochezia, (3) peripheral circulatory failure due to OB, manifested as increased heart rate (higher than 100 times per minute) , decreased blood pressure (systolic blood pressure lower than 90 mmHg and diastolic blood pressure lower than 60 mmHg), (4) with adequate fluid replacement or blood transfusion, central venous pressure decreased, or urine output was low or absent, or red blood cells (RBC), Hb, and hematocrit decreased sharply, and (5) with adequate fluid replacement and urine output, plasma urea nitrogen continued to rise. Active OB was considered relieved if all the above five situations disappeared. If Hb was 80–100 g/L, and/or ECOG = 2, or older than 70 years old, or with active OB before systemic therapy, the chemotherapy dose was reduced by 25%. The systemic anti-cancer regimen was determined by doctors based on patients’ state according to guidelines for gastric cancer. The criterion for treatment delay was that the treatment cycle was postponed by more than 7 days.

All patients received computed tomography (CT), magnetic resonance imaging (MRI), positron emission computed tomography (PET)/CT scans to evaluate treatment efficacy every 6–8 weeks. The response to treatment was evaluated as complete response (CR), partial response (PR), stable disease (SD), and progressive disease (PD) according to the Response Evaluation Criteria in Solid Tumors (RECIST) version 1.1 by clinicians. The objective response rate (ORR) was defined as the rate of CR and PR, and the disease control rate (DCR) was defined as the proportion of CR, PR, and SD. The progression-free survival (PFS) was defined as the time from the beginning of systemic anti-cancer treatment to disease progression or death from any cause, whichever happened first. The overall survival (OS) was defined as the time from the beginning of systemic anti-cancer treatment to death from any cause. TRAEs were evaluated according to the National Cancer Institute Common Toxicity Criteria (NCI-CTC) version 4.0. Patients were followed up by nurses every three months until death or loss to follow-up.

### Statistical analysis

SPSS version 25.0 (IBM, New York, USA) was applied to analyze the data, and differences were considered statistically significant when the two-sided *P* values were less than 0.05. We assessed quantitative variables by Wilcoxon's non-parametric test and categorical variables by the chi-square or Fisher's exact test. A binary logistic regression model was used to evaluate factors that independently influenced POBPST. The PFS and OS were estimated by the Kaplan–Meier method with the log-rank test. The Cox proportional hazard regression model was used to calculate the hazard ratio (HR) with corresponding 95% CIs.

## Results

### Patients characteristics

**Fig. 1:**
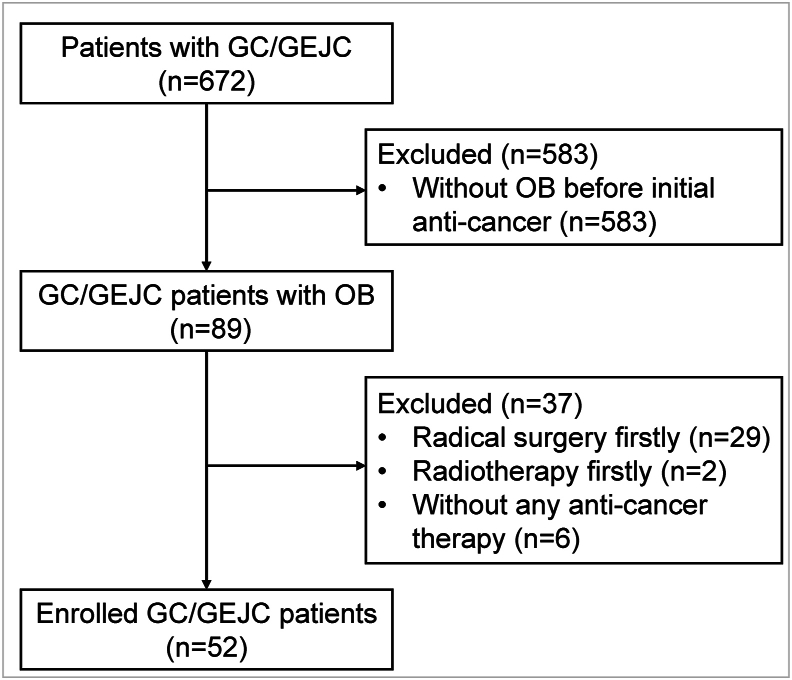
Flowchart of patient selection. GC/GEJC, gastric cancer or gastroesophageal junction cancer; OB, overt bleeding.

A total of 672 GC/GEJC patients were reviewed, and 52 patients with OB were enrolled finally (Fig. 1). The characteristics of enrolled patients are presented in Table 1. In the whole population, 75.0% of patients were diagnosed with stage IV GC/GEJC, and 25.0% were at stage III. Six patients were confirmed as human epidermal growth factor receptor 2 (Her-2) positive (scored as 3+ on immunohistochemistry or fluorescence *in situ* hybridization positive). Twenty-four patients received four mismatch repair (MMR) protein staining, and six patients were confirmed as MMR-deficient (dMMR).

**Table 1:** Characteristics of enrolled patients (*N* ​= ​52).

Clinical characteristics	*n* (%)
**Sex**	
Male	34 (65.4)
Female	18 (34.6)
**Age (years, median, range)**	66 (47–85)
**Age (years)**	
< 60	8 (15.4)
≥ 60	44 (84.6)
**ECOG-PS**	
0 or 1	45 (86.5)
2	7 (13.5)
**BMI (kg/m**^**2**^**)**	
< 18.4	7 (13.5)
18.5–23.9	32 (61.5)
≥ 24.0	13 (25.0)
**Primary tumor location**	
Not GEJC	39 (75.0)
GEJC	13 (25.0)
**Tumor histology**	
MDA/HDA	15 (28.8)
LDA/SRC	37 (71.2)
**Cancer stage (AJCC 8th)**	
Stage III	13 (25.0)
Stage IV	39 (75.0)
**Tstage**	
T3	3 (5.8)
T4	41 (78.8)
NA	8 (15.4)

ECOG-PS, Eastern Corporative Oncology Group Performance Status; BMI, body mass index; GEJC, gastroesophageal junction cancer; LDA, lowly differentiated adenocarcinoma; MDA, moderately differentiated adenocarcinoma; HAD, highly differentiated adenocarcinoma; SRC, signet-ring carcinoma; AJCC eighth, American Joint Committee on Cancer eighth revision; NA, not appliable.

### Presentation of OB and hemostatic treatment

The most initial presentation of OB was melena (*n* = 36, 69.2%), followed by 8 patients (15.4%) presented with both melena and haematemesis, 4 patients (7.7%) with only haematemesis, 1 patient (1.9%) with hematochezia only, and 3 patients (5.8%) were diagnosed as OB by gastroscopy. The median minimum Hb for all patients was 69 (range, 38–118) g/L (Table 2).

**Table 2:** Presentation of OB and hemostatic treatment (*N* ​= ​52).

Clinical characteristics	*n* (%)
**Initial presentation of OB**	
Melena	36 (69.2)
Haematemesis	4 (7.7)
Hematochezia	1 (1.9)
Melena and haematemesis	8 (15.4)
Gastroscopy visible bleeding	3 (5.8)
**With active OB in one month before anti-cancer therapy**	
Yes	19 (36.5)
No	33 (63.5)
**Laboratory data at the time of bleeding**	
Median minimum Hb (g/L, median, range)	69 (38–118)
Median minimum RBCs ( ​× ​10^12^/L, median, range)	2.85 (1.45–5.32)
**Hemostasis therapy**	
Endoscopic hemostasis	2 (3.8)
Only drug treatment	50 (96.2)
**RBCs transfusion (Units)**	
0	26 (50.0)
1-4	18 (34.6)
5-8	8 (15.4)
**Median Hb with three days before anti-cancer therapy (g/L, median, range)**	94.5 (66–120)

OB, overt bleeding; Hb, hemoglobin; RBCs, red blood cells.

Within one month prior to anti-cancer therapy, 19 patients (36.5%) experienced active OB. Two of them received gastroscopic hemostasis, and the other 17 received conservative drug treatment, such as hemostatic drugs, gastric mucosal protective agents, and proton pump inhibitors. All these patients with active OB achieved hemodynamic stabilization. The other 33 patients with non-active OB were treated with gastric mucosal protective agents and/or proton pump inhibitors. Half of these patients received RBCs transfusion. The median RBCs transfusion was 4 (range, 1–8) units.

### Systemic treatment regimens for the patients

**Fig. 2:**
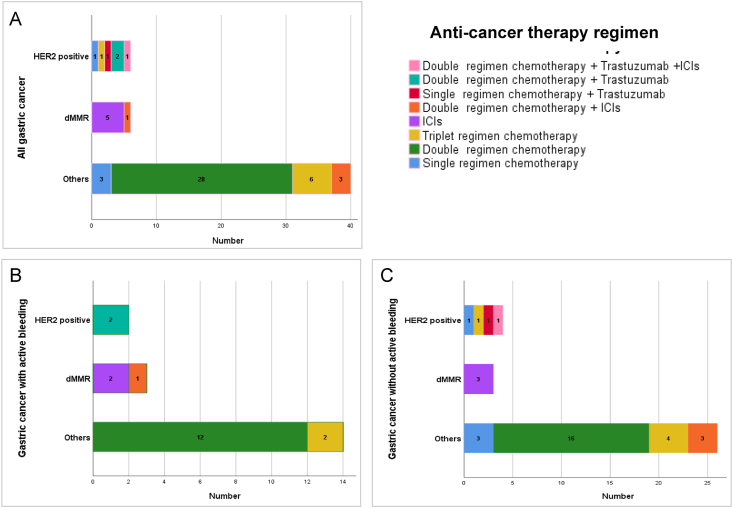
Anti-cancer therapy regimen for gastric cancer patients. ICIs, immune checkpoint inhibitors; HER2, human epidermal growth factor receptor 2.

The median Hb before anti-cancer therapy was 94.5 (66–120) g/L. The median interval time between the time when active OB was controlled and initiation of anti-cancer therapy was 16 days (range 3–25 days) for the 19 patients with active OB. Of all patients, 5 patients received ICIs monotherapy, and 47 patients received chemotherapy-based systemic therapy, including chemotherapy, chemotherapy plus anti-Her-2 therapy and/or ICIs, fluorouracil monotherapy (Fig. 2A). And 70.2% (33/47) of the patients had a chemotherapy dose reduction by 25%. Three patients (5.8%) experienced treatment delay after the first cycle. The median therapy time was 4 cycles (1–31 cycles).

For the 19 patients experiencing active OB within 1 month prior to anti-cancer therapy, 14 patients (73.7%) received intravenous systemic anticancer regimens, including mFOLFOX6 regimen (*n* = 7), mFLOT regimen (*n* = 2), mFOLFOX6 plus trastuzumab for Her-2 positive patients (*n* = 2), mFOLFOX6 plus programmed death receptor 1 (PD-1) antibody sintilimab for 1 dMMR patient and PD-1 antibody monotherapy for the other 2 dMMR patients (1 chose pembrolizumab and 1 chose sintilimab). The other five patients received SOX regimen (Fig. 2B). The chemotherapy dose for all the patients with active OB was reduced by 25%.

The regimens for the 33 patients with non-active OB were as follows. For the four Her-2 positive patients, case 1 received SOX plus trastuzumab and PD-1 antibody pembrolizumab, case 2 received S-1 plus trastuzumab, case 3 received DCS regimen without trastuzumab because of allergy, and case 4 who was 85-years old received S-1 monotherapy without trastuzumab because of cardiac dysfunction. Three patients who were dMMR received PD-1 antibody monotherapy (1 chose pembrolizumab and 2 chose sintilimab). For the 26 patients who were neither Her-2 positive nor dMMR, most received doublet regimen chemotherapy, including SOX regimen (*n* = 5), mFOLFOX6 (*n* = 9), DS regimen (*n* = 2). Besides, four patients received DCS regimen, and three patients received SOX plus PD-1 antibody sintilimab, and three patients received S-1 monotherapy (Fig. 2C). Among the 33 patients, 16 patients had a dose reduction by 25%.

### Efficacy and safety

In the whole population, the radiographic best response rates to anti-cancer therapy were CR 1.9% (1/52), PR 40.4% (21/52), SD 48.1% (25/52) and PD 9.6% (5/52). The ORR and DCR were 42.3% and 90.4% respectively. Fourteen (26.9%) patients received radical surgery after anti-cancer therapy. Chi-square tests showed that there were no factors associated with the tumor response to therapy (Supplementary File 3). The median Hb was 105 (74–125) g/L a month after anti-cancer therapy.

**Fig. 3:**
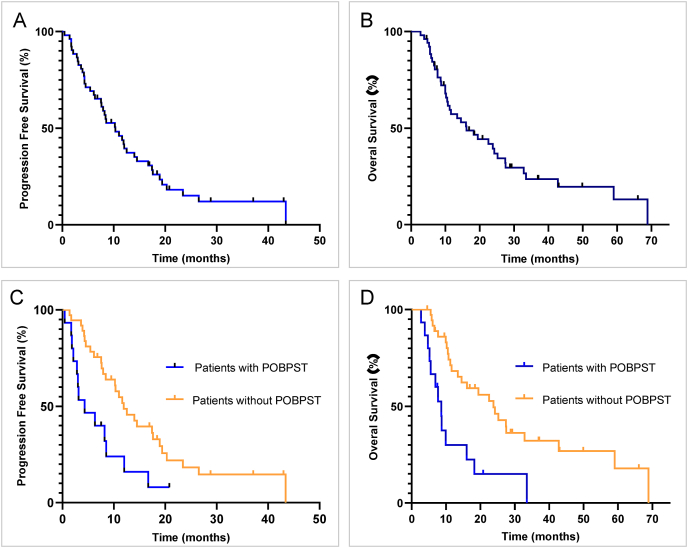
Kaplan Meier curves of progression-free survival and overall survival. POBPST, persistent overt bleeding post systemic therapy.

Until the data cutoff (February 28, 2025), the median follow-up time was 37.1 months (28.6–45.6 months). A total of 42 patients (80.8%) progressed in first-line therapy, and 38 (73.1%) deaths occurred. The mPFS and mOS were 10.3 months (95% CI, 6.6–14.0 months) and 16.1 months (95% CI, 8.3–23.9 months) for the whole population respectively (Fig. 3A and B). The rate of POBPST within 30-days was 28.8%. The mPFS and mOS were worse for the patients with POBPST than the patients without POBPST (mPFS: 4.3 months [95% CI, 0.1–8.5 months] versus 11.9 months [95% CI, 7.8–16.0 months], HR = 2.380, 95% CI, 1.210–4.684, *P* = 0.012; mOS: 8.6 months [95% CI, 5.7–11.5 months] versus 23.8 months [95% CI, 16.3–31.3months], HR = 3.259, 95% CI, 1.607–6.609, *P* = 0.001) (Fig. 3C and D).

The incidence rate of grade 3–4 TRAEs was 36.5%. The most common grade 3–4 TRAEs were hematological toxicity (28.8%) and gastrointestinal reaction (11.5%). There was no 30-days mortality.

### Analysis of patients with POBPST

A total of fifteen patients (28.8%) experienced POBPST within 30-days since anti-cancer therapy. Five of them presented with active POBPST, occurring 2–28 days (median 14 days) after anti-cancer therapy. The POBPST rate was 31.6% (6/19) and 27.3% (9/33) for patients with active OB and non-active OB before systemic anti-cancer therapy respectively, without statistical significance. The characteristics, manifestation and treatment detail were presented in Table 3.

**Table 3:** Details for patients with 30-day POBPST.

Characteristics	POBPST patients		
	Total	Active POBPST	Non-active POBPST
	(*n* ​= ​15)	(*n* ​= ​5)	(*n* ​= ​10)
**Interval between anti-cancer therapy and POBPST (d, median, range)**	NA	14 (2–28)	NA
**With active OB in one month before anti-cancer therapy**			
Yes	6 (40.0%)	3 (60.0%)	3 (30.0%)
No	9 (60.0%)	2 (40.0%)	7 (70.0%)
**Best response to anti-cancer therapy**			
CR/PR	1 (6.7%)	0	1 (10.0%)
SD	10 (66.7%)	4 (80.0%)	6 (60.0%)
PD	4 (26.7%)	1 (20.0%)	3 (30.0%)
**Presentation of POBPST**			
Melena	11 (73.3%)	2 (40.0%)	9 (90.0%)
Haematemesis	2 (13.3%)	1 (20.0%)	1 (10.0%)
Hematochezia	2 (13.3%)	2 (40.0%)	0
**Shock**			
Yes	2 (13.3%)	2 (40.0%)	0
No	13 (86.7%)	3 (60.0%)	10 (100.0%)
**Laboratory data**			
Median minimum Hb (g/L, median, range)	71 (46–109)	54 (46–71)	80 (59–109)
Median minimum RBCs ( ​× ​10^12^/L, median, range)	2.60 (1.45–3.90)	1.96 (1.45–2.63)	2.83 (1.50–3.90)
**Hemostasis therapy**			
Transarterial embolization	2 (13.3%)	2 (40.0%)	0
Conservative drug treatment	13 (86.7%)	3 (60.0%)	10 (100.0%)
**RBCs transfusion (Units)**			
0	8 (53.3%)	0	8 (80.0%)
1-4	5 (33.3%)	3 (60.0%)	2 (20.0%)
5-8	2 (13.3%)	2 (40.0%)	0
**Hemostasis outcome**			
Success	8 (53.3%)	4 (80.0%)	4 (40.0%)
Failure	7 (46.7%)	1 (20.0%)	6 (60.0%)
**Subsequent anti-cancer therapy**			
Chemotherapy/ICIs	8 (53.3%)	2 (40.0%)	6 (60.0%)
Radiotherapy ​± ​chemotherapy	2 (13.3%)	1 (20.0%)	1 (10.0%)
Chemotherapy ​+ ​radical surgery	2 (13.3%)	1 (20.0%)	1 (10.0%)
None	3 (20.0%)	1 (20.0%)	2 (20.0%)

POBPST, persistent overt bleeding post systematic therapy; OB, overt bleeding; Hb, hemoglobin; RBCs, red blood cells; ICIs, immune checkpoint inhibitors; NA, not appliable.

Of the five active POBPST, case 1 to 3 had experienced active OB before systemic anti-cancer therapy, and received mFOLFOX6 regimen. After POBPST happened, case 1 and case 2 got hemostasis through conservative drug treatment, followed by chemotherapy, chemotherapy and radical surgery, respectively. Case 3 had received transarterial embolization twice to stop POBPST, but he continued to disease progression and bleed, and unable to receive anti-cancer therapy again, so he had to receive palliative supportive care and RBCs transfusion when it was necessary without oral nutrition until death nine months later. Case 4 got hemostasis after transarterial embolization and then received chemotherapy. Case 5 stopped bleeding after conservative drug treatment, and received radiotherapy.

Of the ten non-active POBPST, case 1 to 3 underwent active OB before systemic anti-cancer therapy. Case 1 had received mFOLFOX6 regimen with a best response as SD and continued the regimen chemotherapy after POBPST. Case 2 and case 3 were dMMR and received PD-1 antibody therapy. Case 2 got hemostasis with a best response as SD and received treatment continuously. Case 3 got a best response as PD and failed to stop POBPST, but received PD-1 antibody and cytotoxic T-lymphocyte-associated protein 4 (CTLA4) inhibitor. Case 4 and case 5 got hemostasis and then received chemotherapy, chemotherapy followed by radical surgery respectively. Case 6 to 8 couldn't stop POBPST after conservative drug treatment, but it didn't affect the anti-cancer treatment (case 6 and case 7 received chemotherapy, and case 8 received chemotherapy and radiotherapy). Case 9 and case 10 failed to stop bleeding so that they didn't receive any other anti-cancer therapy because of recurrent severe anemia after first-line treatment resistance.

Chi-square tests showed that MBI scores < 75 (60.0% [9/15] vs. 8.1% [3/37], *P* < 0.001), with a best response as PD/SD (PD 26.7% [4/15] vs. 2.7% [1/37], SD 66.7% [10/15] vs. 40.5% [15/37], *P* < 0.001) were risk factors for POBPST (Table 4). Active OB before anti-cancer therapy wasn't a risk factor for POBPST (40.0% [6/15] vs 35.1% [13/37], *P* = 0.741). Grade 3–4 TRAEs (13.3% [2/15] vs. 45.9% [17/37], *P* = 0.031) was protective factor. After adjusting for factors whose P value less than 0.1, logistic regression analysis showed that independent, positive influencing factors for POBPST were MBI scores < 75 and with a best response as PD/SD (Table 4). Wilcoxon's Rank Sum test showed that difference of MBI scores pre and post anti-cancer therapy exhibited a non-normal distribution. The median MBI score for patients with POBPST was decreased more than that for patients without POBPST after anti-cancer therapy with statistically difference (15 vs. 0, *P* = 0.001).

**Table 4:** Chi-square test and binary logistic analysis of factors related to 30-day POBPST.

Characteristics	Chi-square test			Binary logistic analysis	
	Patients without POBPST	Patients with POBPST	*P* value	HR (95% CI)	*P* value
	(*n* ​= ​37)	(*n* ​= ​15)			
**Sex**					
Male	24 (64.9%)	10 (66.7%)	0.902		
Female	13 (35.1%)	5 (33.3%)			
**Age (years)**					
< 60	6 (16.2%)	2 (13.3%)	1.000		
≥ 60	31 (83.8%)	13 (86.7%)			
**ECOG-PS**					
0 or 1	33 (89.2%)	12 (80.0%)	0.397		
2	4 (10.8%)	3 (20.0%)			
**BMI (kg/m**^**2**^**)**					
< 18.4	3 (8.1%)	4 (26.7%)	0.202		
18.5–23.9	25 (67.6%)	7 (46.7%)			
≥ 24.0	9 (24.3%)	4 (26.7%)			
**Morse fall scale assessment**					
Low-risk	22 (59.5%)	5 (33.3%)	0.088	Ref	
Medium-high risk	15 (40.5%)	10 (66.7%)		0.470 (0.038–5.798)	0.556
**MBI scores assessment**					
< 75	3 (8.1%)	9 (60.0%)	< 0.001	78.298 (2.588–2369.186)	0.012
≥ 75	34 (91.9%)	6 (40.0%)		Ref	
**Primary tumor location**					
Body	13 (35.1%)	7 (46.7%)	0.794		
Pylorus	14 (37.8%)	5 (33.3%)			
Cardia	10 (27.0%)	3 (20.0%)			
**Tumor histology**					
MDA/HDA	10 (27.0%)	5 (33.3%)	0.649		
LDA/SRC	27 (73.0%)	10 (66.7%)			
**Cancer stage (AJCC 8th)**					
Stage III	7 (18.9%)	6 (40.0%)	0.112		
Stage IV	30 (81.1%)	9 (60.0%)			
**T Stage**					
T3	2 (5.4%)	1 (6.7%)	0.122		
T4	27 (73.0%)	14 (93.3%)			
NA	8 (21.6%)	0			
**With active OB in one month prior to anti-cancer therapy**					
Yes	13 (35.1%)	6 (40.0%)	0.741		
No	24 (64.9%)	9 (60.0%)			
**RBCs transfusion in one month before anti-cancer therapy**					
5 units	31 (83.8%)	13 (86.7%)	1.000		
≥ ​5 units	6 (16.2%)	2 (13.3%)			
**Hb before anti-cancer treatment**					
< ​100 g/L	21 (56.8%)	11 (73.3%)	0.352		
≥ ​100 g/L	16 (43.2%)	4 (26.7%)			
**Number of chemotherapy drugs**					
None or one	7 (18.9%)	3 (20.0%)	1.000		
Two	25 (67.6%)	10 (66.7%)			
Three	5 (13.5%)	2 (13.3%)			
**Best response to anti-cancer therapy**					
CR/PR	21 (56.8%)	1 (6.7%)	< 0.001	Ref	
SD	15 (40.5%)	10 (66.7%)		20.237 (1.178–347.743)	0.038
PD	1 (2.7%)	4 (26.7%)		49.458 (1.489–1643.042)	0.029
**Grade 3–4 TRAEs**					
Yes	17 (45.9%)	2 (13.3%)	0.031	Ref	
No	20 (54.1%)	13 (86.7%)		10.085 (0.615–165.381)	0.105

POBPST, persistent overt bleeding post systematic therapy; HR, hazard ratio; BMI, Body Mass Index; ECOG-PS, Eastern Corporative Oncology Group Performance Status; MBI, Modified Barthel Index; LDA, lowly differentiated adenocarcinoma; MDA, moderately differentiated adenocarcinoma; HAD, highly differentiated adenocarcinoma; SRC, signet-ring carcinoma; AJCC eighth, American Joint Committee on Cancer eighth revision; NA, not appliable; OB, overt bleeding; RBCs, red blood cells; Hb, hemoglobin; CR, complete response; PR, partial response; SD, stable disease; PD, progressive disease; TRAEs, treatment related adverse events; Ref, reference; CI, confidence interval.

OB was stopped after systemic anti-cancer treatment for 71.2% (37/52) of patients. Some of those patients had achieved long time survival. For example, one 59 years old female patient, who was diagnosed as staged IV, lung metastasis, HER2 positive, and experienced active OB with a lowest Hb 59 g/L, got PR from first line anti-cancer treatment with a PFS 18.9 months. Though disease relapsed, she benefitted from posterior line therapy and survived well until last follow-up with an OS 66.1+ months. Another 62 years old male patient went to hospital due to acute gastric bleeding combined with severe anemia (the lowest Hb = 65 g/L) and diagnosed as staged IV with retroperitoneal lymph node metastasis, and got PR from first line anti-cancer treatment followed by radical surgery with a disease-free survival 20.4 months and OS 68.9 months. A 64-years old male patient couldn't receive radical surgery because gastric cancer (HER2 negative and proficient mismatch repair) invaded liver (the lowest Hb = 53 g/L), so he received systemic anti-cancer treatment. The PFS for first line was 43.4 months, and he was still alive with an OS 49.9+ months up to the last follow up time.

## Discussion

### Main findings

**Fig. 4:**
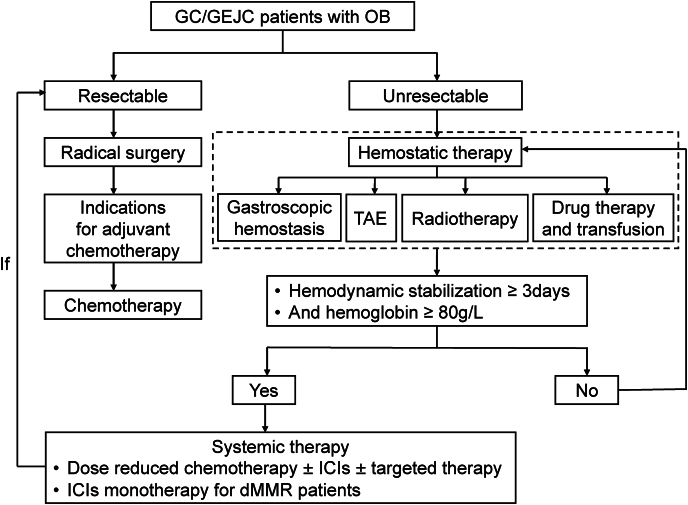
Treatment scheme for gastric cancer (GC) / gastroesophageal junction cancer (GEJC) patients with overt bleeding (OB). ICIs, immune checkpoint inhibitors; TAE, transcatheter arterial embolization.

This study demonstrated that systemic anti-cancer therapy was feasible for patients with advanced or metastatic GC/GEJC with OB, though such patients are often excluded from clinical trials due to concerns over bleeding risk and poor treatment tolerance (Fig. 4). In this cohort, 70.2% of patients received a 25% dose reduction of chemotherapy to minimize complications. The overall ORR and DCR were 42.3% and 90.4%, respectively, suggesting that reduced-dose systemic therapy may be both safe and effective in this vulnerable population.

These findings are consistent with prior studies, such as the GO2 trial, which found that reduced chemotherapy doses (by 20% to 40%) did not compromise PFS in frail patients.^20^ Similarly, real-world data from patients with lung squamous cell carcinoma showed comparable outcomes between standard and reduced-dose chemotherapy, with fewer AEs in the dose-reduction group.^21^

In this study, the median interval between initiating systemic therapy and the occurrence of POBPST was 14 days—longer than previously reported^4^—likely because all enrolled patients achieved hemostasis before treatment and were in relatively stable condition. The incidence of POBPST was 28.8%, with nearly three-quarters of these events attributed to unresolved primary bleeding rather than disease progression.

The study also identified that patients with pre-treatment ADL-MBI scores below 75 were at greater risk for POBPST, highlighting the importance of functional status in predicting treatment tolerance. Poor nutritional status, tumor burden, and comorbidities may contribute to rebleeding in this population. These findings support earlier research that linked higher ADL scores with better treatment outcomes and reinforce the relevance of comprehensive pre-treatment assessments.^22^, ^23^

Interestingly, patients who experienced grade 3–4 TRAEs had a lower incidence of POBPST, likely because their subsequent treatment cycles were adjusted (i.e., further dose reduction and delay), coupled with supportive care measures such as nutritional support and prophylactic hemostasis.

Despite these encouraging findings, several patients failed to achieve sustained hemostasis, making subsequent anti-cancer therapy difficult to implement. Local hemostatic interventions—endoscopic, radiologic, or radiation-based—were often only temporarily effective, and frail patients who re-bled during treatment had poorer survival.

### Implications for nursing practice and research

This study underscores the essential role of oncology nurses in managing patients with GC/GEJC and OB, especially in real-world clinical settings. Nurses were instrumental in assessing treatment tolerance, functional status, and AEs, guiding timely dose modifications, and coordinating supportive interventions.^24^, ^25^

Nursing responsibilities in this context include monitoring chemotherapy-related side effects, providing education on self-care and symptom management, offering emotional support, and delivering physical care. Evidence from previous studies supports the effectiveness of specialized nursing interventions—such as holistic or intensive nursing care—in improving psychological well-being, reducing malnutrition, and enhancing overall quality of life in patients with advanced cancer.^26^, ^27^, ^28^

Importantly, this study suggests that structured nursing assessment tools, such as ADL-MBI, can help identify patients at risk for poor outcomes, allowing for tailored interventions. Nurses can play a proactive role in early detection of rebleeding, treatment planning, and coordination of multidisciplinary care involving oncology, nutrition, interventional radiology, and palliative services.

Future research should further explore nursing-led models of care, including early nutritional support, pre-treatment functional assessments, and rehabilitation programs. Developing standardized nursing care pathways for this patient population could improve treatment safety, quality of life, and long-term outcomes.

### Limitations

This study has several limitations. First, it was a single-center retrospective analysis with a relatively small sample size, largely due to the rarity of systemic anti-cancer treatment in GC/GEJC patients with OB. The limited sample may have reduced statistical power and generalizability. Second, the treatment regimens and durations were heterogeneous, reflecting the real-world setting but also introducing variability that may affect interpretation of outcomes. Additionally, POBPST, as a clinical endpoint, is influenced by multiple factors beyond systemic therapy, including tumor characteristics, comorbidities, and local interventions. Future large-scale, multicenter prospective studies are warranted to validate these findings, identify optimal treatment strategies for this high-risk group, and develop standardized protocols. Importantly, the role of oncology nursing in treatment decision-making, patient education, and supportive care deserves further investigation to enhance both safety and quality of care in this population.

## Conclusions

This study demonstrated that appropriately dose-adjusted systemic anti-cancer therapy is a feasible and generally tolerable option for patients with unresectable GC/GEJC presenting with OB. Among clinical predictors, baseline functional status emerged as a key factor associated with the risk of POBPST, which was linked to significantly worse survival outcomes. These findings underscore the importance of individualized treatment planning and highlight the critical role of oncology nursing in patient assessment, supportive care, and multidisciplinary coordination. With adequate hemostatic control and tailored supportive strategies, systemic therapy can remain a viable therapeutic avenue for this clinically challenging population.

## CRediT authorship contribution statement

**Yanhong Yao**: Conceptualization, Methodology, Investigation, Formal Analysis, Writing - Original Draft. **Zhentao Liu**: Supervision, Visualization. **Hua Zhang**: Formal Analysis, Validation. **Xinhua Shi**: Data Curation, Investigation. **Fangfang Huang**: Resources, Investigation. **Yi Zhang**: Validation, Writing - Review & Editing. **Lu Chen**: Data Curation, Investigation. **Yanyan Shi**: Formal Analysis. **Baoshan Cao**: Conceptualization, Methodology, Resources, Supervision, Writing - Review & Editing. All authors have read and approved the final manuscript.

## Ethics statement

This study has been approved by the Ethics Committee of Peking University Third Hospital (IRB No. 00006761-M2023544) and was conducted in accordance with the 1964 Helsinki Declaration and its later amendments or comparable ethical standards. Informed consent was waived by our Institutional Review Board because of the retrospective nature of our study.

## Data availability statement

The datasets used during the current study are available from the corresponding author upon reasonable request.

## Declaration of generative AI and AI-assisted technologies in the writing process

No AI tools/services were used during the preparation of this work.

## Funding

This study was supported by Key Clinical Projects of Peking University Third Hospital (Grant No. BYSYZD2024018). The funders had no role in considering the study design or in the collection, analysis, interpretation of data, writing of the report, or decision to submit the article for publication.

## Declaration of competing interest

The authors declare no conflict of interest.
