# Supplementary material for: Feasibility of systemic therapy in unresectable gastric/gastroesophageal junction cancer with overt bleeding
Source: Asia Pac J Oncol Nurs. 2025 Jul 7;12:100750. doi: 10.1016/j.apjon.2025.100750 (PMC12310399; doi:10.1016/j.apjon.2025.100750)
Supplement: Multimedia component 1 [file mmc1.docx]

Supplementary File 1

| 项目 | 评分标准 | 评分 |
| --- | --- | --- |
| 吃饭 | 0分：依赖他人 |  |
|  | 5分：需要部分帮助（夹菜、盛饭、切面包） |  |
|  | 10分：全面自理 |  |
| 洗澡 | 0分：依赖他人 |  |
|  | 5分：全面自理 |  |
| 修饰 | 0分：需要帮助 |  |
|  | 5分：独立洗脸、梳头、刷牙、剃须 |  |
| 穿衣 | 0分：依赖他人 |  |
|  | 5分：需一般帮助 |  |
|  | 10分：自理（系、开纽扣，开、闭拉锁和穿鞋等） |  |
| 入厕 | 0分：依赖他人 |  |
|  | 5分：需部分帮助 |  |
|  | 10分：全面自理 |  |
| 大便 | 0分：失禁或昏迷 |  |
|  | 5分：偶尔失禁（每周＜1次） |  |
|  | 10分：能控制 |  |
| 小便 | 0分：失禁或昏迷或由他人导尿 |  |
|  | 5分：偶尔失禁（每24h＜1次，每周＞1次） |  |
|  | 10分：能控制 |  |
| 床椅转移 | 0分：依赖他人，不能坐 |  |
|  | 5分：能坐，但需大量（2人）帮助 |  |
|  | 10分：需少量（1人）帮助或指导 |  |
|  | 15分：全面自理 |  |
| 行走 | 0分：不能走 |  |
|  | 5分：在轮椅上独立行动 |  |
|  | 10分：需1人帮助（体力或语言督导） |  |
|  | 15分：独自步行 (可用辅助器) |  |
| 上下楼梯 | 0分：不能 |  |
|  | 5分：需帮助 |  |
|  | 10分：自理 |  |
| 总分 | |  |
| 评估人员 | |  |
| 评估结果说明： 0-20分：极严重功能缺陷；25-45分：严重功能缺陷；50-70分：中度功能缺陷；75-95分：轻度功能缺陷；100分：ADL能自理。 | | |

ADL改良 Barthel Index（MBI）评估量表

姓名： 性别： 床号： 住院号： 日期：

Supplementary File 2

Morse跌倒风险评估量表

姓名： 性别： 床号： 住院号： 日期：

| 评估内容 | 评分标准 | 评分 |
| --- | --- | --- |
| 3个月内有无跌倒史 | 没有：0分 |  |
|  | 有：25分 |  |
| 有无超过1个医学诊断 | 没有：0分 |  |
|  | 有：15分 |  |
| 有无使用助行器具 | 没有需要：0分 |  |
|  | 卧床且不能主动转移：0分 |  |
|  | 由护士或其他人扶行：0分 |  |
|  | 使用拐杖/手杖：15分 |  |
|  | 使用四角叉：15分 |  |
|  | 依扶家具：30分 |  |
| 有无静脉输液/留置套管针 | 没有：0分 |  |
|  | 有：20分 |  |
| 步态 | 正常：0分 |  |
|  | 卧床且不能主动转移：0分 |  |
|  | 轮椅代步：0分 |  |
|  | 虚弱无力：10分 |  |
|  | 功能受损：20分 |  |
| 认知能力 | 正确了解自己的能力：0分 |  |
|  | 高估自己的能力：15分 |  |
|  | 忘记自己受限制：15分 |  |
| 总分 | |  |
| 评估人员 | |  |
| 评估结果说明：  评分0-24分：低度跌倒风险  25-44分：中度跌倒风险  ≥45分：高度跌倒风险 | | |

Supplementary File 3.

Chi-square test of factors related to tumor response to anti-cancer therapy

| Characteristics | CR/PR | SD | PD | *P* value |
| --- | --- | --- | --- | --- |
|  | (*n* = 22) | (*n* = 25) | (*n* = 5) |  |
| Sex |  |  |  |  |
| Male | 14 (63.6%) | 17 (68.0%) | 3 (60.0%) | 0.918 |
| Female | 8 (36.4%) | 8 (32.0%) | 2 (40.0%) |  |
| Age (years) |  |  |  |  |
| < 60 | 5 (22.7%) | 3 (12.0%) | 0 | 0.511 |
| ≥ 60 | 17 (77.3%) | 22 (88.0%) | 5 (100.0%) |  |
| ECOG-PS |  |  |  |  |
| 0 or1 | 20 (90.9%) | 21 (84.0%) | 4 (80.0%) | 0.596 |
| 2 | 2 (9.1%) | 4 (16.0%) | 1 (20.0%) |  |
| BMI (kg/m^2^) |  |  |  |  |
| < 18.4 | 1 (4.5%) | 5 (20.0%) | 1 (20.0%) | 0.373 |
| 18.5-23.9 | 15 (68.2%) | 15 (60.0%) | 2 (40.0%) |  |
| ≥ 24.0 | 6 (27.3%) | 5 (20.0%) | 2 (40.0%) |  |
| Morse Fall Scale^a^ assessment | |  |  |  |
| Low-risk | 13 (59.1%) | 12 (48.0%) | 2 (40.0%) | 0.656 |
| Medium-high risk | 9 (40.9%) | 13 (52.0%) | 3 (60.0%) |  |
| MBI scores assessment |  |  |  |  |
| < 75 | 3 (13.6%) | 6 (24.0%) | 3 (60.0%) | 0.087 |
| ≥ 75 | 19 (86.4%) | 19 (76.0%) | 2 (40.0%) |  |
| Primary tumor location |  |  |  |  |
| Body | 5 (22.7%) | 6 (24.0%) | 2 (40.0%) | 0.938 |
| Pylorus | 8 (36.4%) | 10 (40.0%) | 2 (40.0%) |  |
| Cardia | 9 (40.9%) | 9 (36.0%) | 1 (20.0%) |  |
| Tumor histology |  |  |  |  |
| MDA/HDA | 7 (31.8%) | 7 (28.0%) | 1 (20.0%) | 1.000 |
| LDA/SRC | 15 (68.2%) | 18 (72.0%) | 4 (80.0%) |  |
| Gastric cancer subtype |  |  |  |  |
| HER2 positive | 3 (13.6%) | 2 (8.0%) | 1 (20.0%) | 0.575 |
| dMMR | 3 (13.6%) | 2 (8.0%) | 1 (20.0%) |  |
| Others | 16 (72.7%) | 21 (84.0%) | 3 (60.0%) |  |
| Cancer stage (AJCC 8^th^) | |  |  |  |
| Stage III | 7 (31.8%) | 5 (20.0%) | 1 (20.0%) | 0.723 |
| Stage IV | 15 (68.2%) | 20 (80.0%) | 4 (80.0%) |  |
| T stage |  |  |  |  |
| T3 | 2 (9.1%) | 0 | 1 (20.0%) | 0.242 |
| T4 | 17 (77.3%) | 21 (84.0%) | 3 (60.0%) |  |
| NA | 3 (13.6%) | 4 (16.0%) | 1 (20.0%) |  |
| With active OB in one month prior to anti-cancer therapy | | |  |  |
| Yes | 7 (31.8%) | 10 (40.0%) | 2 (40.0%) | 0.914 |
| No | 15 (68.2%) | 15 (60.0%) | 3 (60.0%) |  |
| Hb before anti-cancer treatment | |  |  |  |
| < 100 g/L | 15 (68.2%) | 14 (56.0%) | 3 (60.0%) | 0.772 |
| ≥ 100 g/L | 7 (31.8%)1 | 11 (44.0%) | 2 (40.0%) |  |
| Anti-cancer regimen | |  |  |  |
| ICIs monotherapy | 3 (13.6%) | 1 (4.0%) | 1 (20.0%) | 0.546 |
| Single regimen chemotherapy | 2 (9.1%) | 2 (8.0%) | 1 (20.0%) |  |
| Double regimen chemotherapy ± ICI ± Trastuzumab | 15 (68.2%) | 17 (68.0%) | 3 (60.0%) |  |
| Triplet regimen chemotherapy | 2 (9.1%) | 5 (20.0%) | 0 |  |

CR, complete response; PR, partial response; SD, stable disease; PD, progressive disease; ECOG-PS, Eastern Corporative Oncology Group Performance Status; BMI, body mass index; GEJC, gastroesophageal junction cancer; LDA, lowly differentiated adenocarcinoma; MDA, moderately differentiated adenocarcinoma; HAD, highly differentiated adenocarcinoma; SRC, signet-ring carcinoma; dMMR, mismatch repair-deficient; AJCC 8^th^, American Joint Committee on Cancer 8th revision; NA, not appliable; OB, overt bleeding; Hb, hemoglobin; RBCs, red blood cells; ICIs, immune checkpoint inhibitors.
